# Crystal structure of 2-[(2*E*)-2-methyl-3-phenyl­prop-2-en-1-yl­idene]-*N*-phenyl­hydrazinecarboxamide

**DOI:** 10.1107/S2056989018018376

**Published:** 2019-01-08

**Authors:** S. R. Saritha, L. Anitha, S. R. Layana, M. Sithambaresan, M. R. Sudarsanakumar

**Affiliations:** a Department of Chemistry, Mahatma Gandhi College, University of Kerala, Thiruvananthapuram 695 004, Kerala, India; b Department of Chemistry, Faculty of Science, Eastern University, Sri Lanka, Chenkalady, Sri Lanka

**Keywords:** crystal structure, semicarbazone, phenyl­hydrazinecarboxamide

## Abstract

The title compound, C_17_H_17_N_3_O, crystallizes with two independent mol­ecules in the asymmetric unit. The semicarbazone moieties of these independent mol­ecules (I and II) are essentially planar [maximum deviation of 0.042 (1) Å in mol­ecule I and 0.041 (1) Å in mol­ecule II], with the terminal phenyl rings twisted away from the mean plane of the semicarbazone moiety, making dihedral angles of 60.26 (8) and 28.76 (9)° in mol­ecule I and 31.07 (9) and 35.45 (8)° in mol­ecule II. The mol­ecules both exhibit an *E* configuration with respect to the C=C and azomethine C=N bonds. In the crystal, two classical N—H⋯O hydrogen-bonding inter­actions are present between the two mol­ecules, forming a centrosymmetric dimer, while a weak C—H⋯O non-classical hydrogen-bonding inter­action, with a donor–acceptor distance of 3.476 (2) Å, inter­connects two neighbouring centrosymmetric dimers to form a cage-like structure. These cage structures are inter­connected by weak C—H⋯π inter­actions with an H⋯π distance of 2.790 Å, forming supra­molecular chains along the *c-*axis direction.

## Abstract 2

The title compound crystallizes with two independent mol­ecules in the asymmetric unit. The semicarbazone moieties of which are essentially planar with the terminal phenyl rings twisted away from them. In the crystal, N—H⋯O hydrogen-bonding inter­actions link the two mol­ecules into a centrosymmetric dimer, with adjacent dimers linked by weak C—H⋯O inter­actions to form a cage-like structure. These cage structures are inter­connected by weak C—H⋯π inter­actions, forming supra­molecular chains along the *c-*axis direction.

## Chemical context   

Semicarbazones are oxygen and nitro­gen contributor ligands whose significance lies in their versatility of mol­ecular sequence, which allows diverse geometries to be obtained. Semicarbazones exhibit amido–iminol tautomerism in solution due to the inter­action of solvent mol­ecules, but generally exist in the amido form in the solid state. The FT–IR and NMR spectra of semicarbazones indicate the existence of a keto form in the solid state that can be confirmed by single crystal X-ray diffraction analysis (Kurup *et al.*, 2011 ▸; Sreekanth *et al.*, 2004 ▸). Biological properties linked to anti­microbial (Siji *et al.*, 2010 ▸) and anti­parasitic (Soares *et al.*, 2011 ▸) effects make semicarbazones important ligands in coordination chemistry. Compared to Gentamycin, a commonly used anti­biotic, *N* ^4^-phenyl­semicarbazone derivatives exhibit moderate anti­bacterial activity at higher concentrations and also show DNA cleavage properties (Layana *et al.*, 2016 ▸). Semicarbazones can function as brilliant ligands in a variety of metal ions (Kala *et al.*, 2007 ▸) and co-ordinate to metal ions either in neutral (Siji *et al.*, 2011 ▸) or in anionic forms (Reena *et al.*, 2008 ▸). Structural studies of many semicarbazones and *N* ^4^-phenyl­semi­carb­azones have been reported and some of them adopt an *E* configuration with respect to the azomethine double bond along with both inter- and intra­molecular hydrogen-bonding inter­actions (Reena *et al.*, 2010 ▸; Layana *et al.*, 2014 ▸, 2018 ▸). Semicarbazones form complexes with a variety of structural features such as monomer, dimer and one-dimensional polymers (Kunnath *et al.*, 2016 ▸). α-Meth­yl*-trans*-cinnamaldehyde, a precursor for the synthesis of α-methyl-*trans*-cinnamaldehyde-*N* ^4^-phenyl­semicarbazone, has significant anti­fungal activity and can self-couple and form complexes with some transition metals (Shreaz *et al.*, 2011 ▸). The diverse structural features and substantial biological applications have prompted us to synthesize a new semicarbazone derived from α-methyl-*trans*-cinnamaldehyde and *N* ^4^-phenyl­semicarbazide.
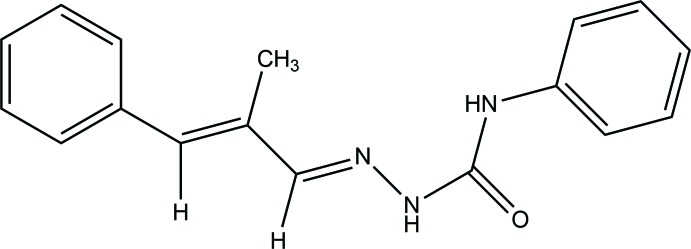


## Structural commentary   

**Figure 1:**
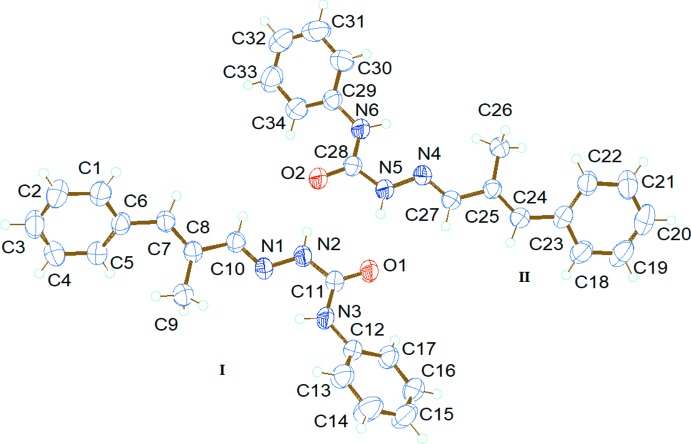
*ORTEP* diagram showing the two mol­ecules in the asymmetric unit, with atom labels and 50% probability displacement ellipsoids.

The title compound crystallizes in the triclinic space group *P* 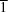 symmetry with two independent mol­ecules, I and II, in the asymmetric unit (Fig. 1 ▸). The semicarbazone units in I and II are essentially planar, with maximum deviations from the least-squares plane of 0.042 (1) Å for N2 in mol­ecule I and 0.041 (1) Å for N4 in mol­ecule II. The terminal phenyl rings in both two mol­ecules are twisted away from the semicarbazone mean plane, making dihedral angles of 60.26 (8) and 28.76 (9)° in mol­ecule I and 31.07 (9) and 35.45 (8)° in mol­ecule II. Both mol­ecules exist in an *E* configuration with respect to the C=C and azomethine C=N bonds. The azomethine C=N and keto C=O bond lengths [1.273 (2) and 1.2269 (17) Å, respectively] in mol­ecule I are shorter than those for mol­ecule II [1.2766 (19) Å and 1.2302 (18) Å respectively]. In contrast, the C=N and C=O bond lengths bond lengths reported for the two independent mol­ecules of 2-benzoyl­pyridine semicarbazone are 1.294 (2) and 1.295 (2) Å and 1.2360 (19) and 1.2390 (19) Å respectively (de Lima *et al.*, 2008 ▸).

## Supra­molecular features   

**Figure 2:**
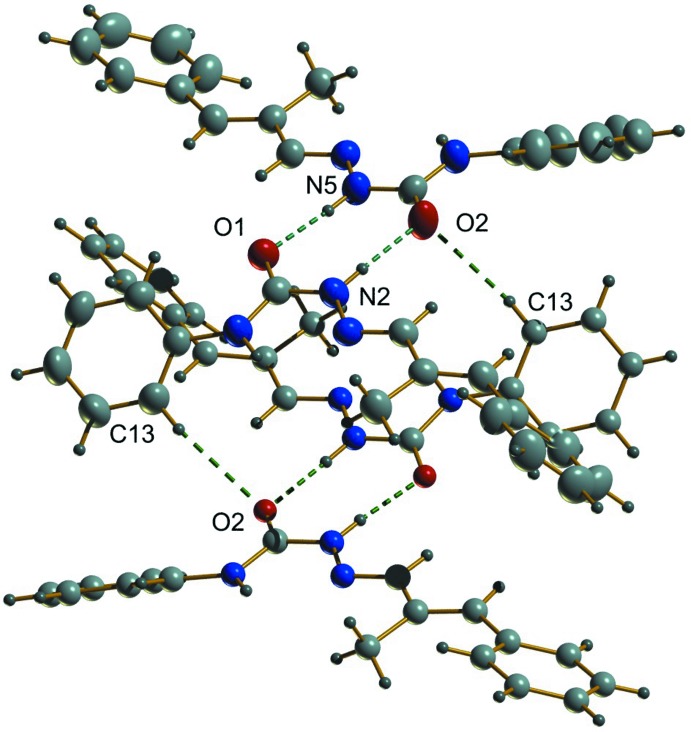
N—H⋯O hydrogen bonds and weak C—H⋯O inter­molecular inter­actions (dashed lines) generating centrosymmetric dimers and a cage-like structure.

**Figure 3:**
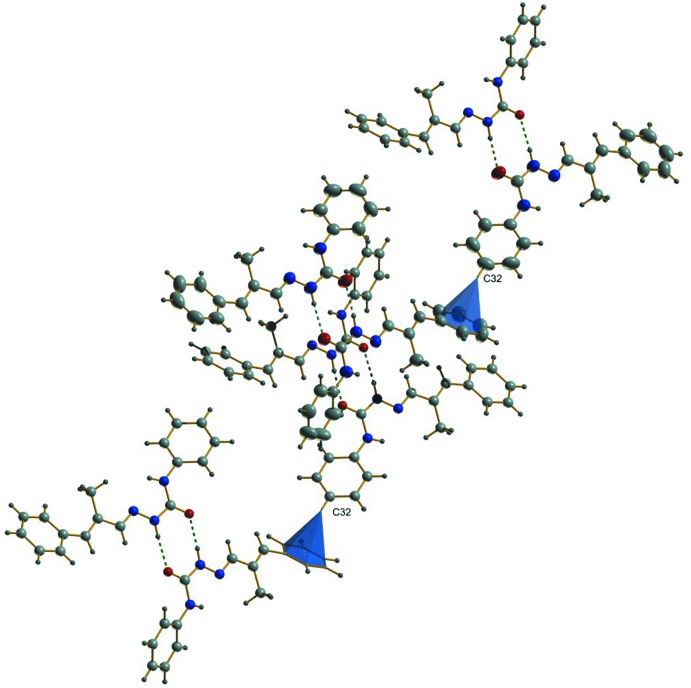
Weak C—H⋯π inter­molecular inter­actions (solid cones), linking the dimeric cage-like structures into a chain along the *c* axis.

**Figure 4:**
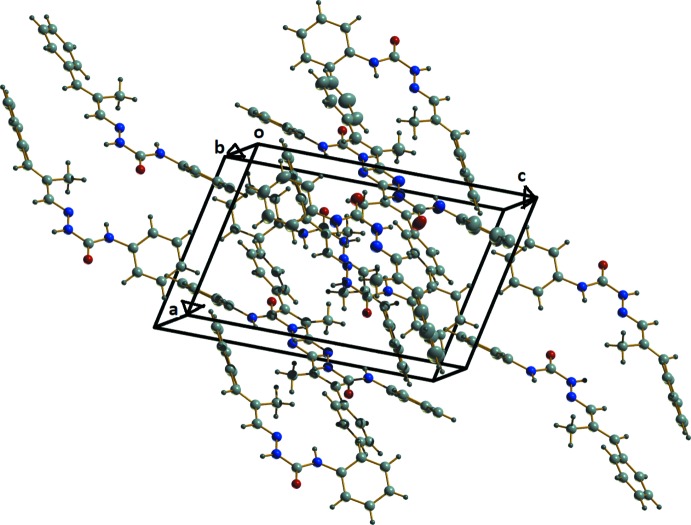
The packing viewed along the *b* axis.

In the crystal, two classical and one non-classical hydrogen-bonding inter­actions are observed. Mol­ecules I and II are linked into centrosymmetric dimers through N2—H2′⋯O2 and N5—H5′⋯O1 hydrogen bonds with *D*⋯*A* distances of 2.808 (2) Å, and 2.8639 (19) Å, respectively (Fig. 2 ▸, Table 1 ▸), while C13—H13⋯O2 inter­actions with a D⋯A distance of 3.476 (2) Å, inter­connect adjacent dimers, creating cage-like structures that are linked by weak C—H⋯π inter­actions into supra­molecular chains along the c-axis direction (Fig. 3 ▸). No significant π–π inter­actions occur. The packing viewed along the *b* axis is shown in Fig. 4 ▸.

### Database survey   

The structure of the title compound has not previously been reported (CSD version 5.39, update of August 2018; Groom *et al.*, 2016 ▸). All geometric parameters in the title compound agree well with those reported in the literature with the C10—N1/C27—N4 [1.273 (2) and 1.2766 (19) Å], N1— N2/N4—N5 [1.3691 (17) and 1.3679 (18) Å] and C11—O1/C28—O2 [1.2269 (17) and 1.2302 (18) Å] bond distances being comparable to those in benzaldehyde-*N* ^4^-phenyl­semi­carb­azone [1.273 (2), 1.369 (2) and 1.225 (2) Å; Layana *et al.*, 2014 ▸] and vanillin-*N*-phenyl­thio­semicarbazone [1.2726 (17), 1.3801 (15) and 1.2404 (15) Å; Layana *et al.*, 2016 ▸]

## Synthesis and crystallization   

Hot ethano­lic solutions of *N* ^4^-phenyl­semicarbazide (0.1512 g, 1 mmol) and α-methyl-*trans*-cinnamaldehyde (0.14 ml, 1 mmol) were mixed and refluxed for about 4 h. Colourless block-shaped crystals of the title compound (yield 83%) were separated by filtration, washed with ethanol and dried over P_4_O_10_ *in vacuo*. Single crystals (m.p. 463±2 K) were obtained by slow evaporation of a 1:1 mixture of ethyl acetate and ethanol.

Analysis calculated: C, 73.03; H, 6.09, N, 15.04%. Found: C, 72.66; H, 6.32; N, 15.29%. Spectrometric data. FT–IR ν_max_ (KBr, cm^−1^): The spectrum of the title compound shows characteristic absorption bands of the main functional groups at IR (ν_max_, cm^−1^): 3379 (^4^NH), 3192 (^2^NH), 1685 (C=O) 3072, 2960 (C—H aromatic), 1591 (C=N), 1029 (N—N). FT–Raman (cm^−1^) 3055 (N—H), 1613 (C=O), 1577 (C=N), 1137 (N—N). ^1^H NMR (400 MHz) (DMSO-*d*_6_, ppm): δ_H_ 2.2 (*s*, 3H, meth­yl), 7–7.5 (*m*, 10H, Ar—H), 6.7 (*s*, 1H, methine), 7.7 (*s*, 1H, azomethine), 8.6 (*s*, 2H, amine), 10.6 (*s*, 1H, iminol H). ^13^C NMR (400 MHz) (DMSO-*d*_6_, ppm): δ_C_ 135.2 (C6), 129.12 (C1 and C5), 128.4 (C2 and C4), 119.6 ppm (C3), 152.9 (C7), 146.4 (C8), 138.9 (C9), 136.5 (C10), 12.9 (C17), 134.3 (C11), 128.5 ppm (C12 and C16), 127.4 ppm (C13 and C15) and 122.4 ppm (C14). UV–visible (200–1000, nm): 268 (π–π*), 342 (*n*–π*).

## Refinement   

Crystal data, data collection and structure refinement details are summarized in Table 2 ▸. Reflections (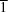 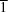
1) and (001) were omitted due to bad agreement. All hydrogen atoms bound to carbon atoms were positioned geometrically with C—H distances of 0.93–0.96 Å and refined as riding, with *U*_iso_(H) = 1.2*U*_eq_(C) or 1.5*U*_eq_(C-meth­yl). The NH hydrogen atoms were located in a difference-Fourier map and refined with N—H restrained to 0.88±0.01 Å.

## Supplementary Material

Crystal structure: contains datablock(s) I. DOI: 10.1107/S2056989018018376/jj2206sup1.cif

Structure factors: contains datablock(s) I. DOI: 10.1107/S2056989018018376/jj2206Isup2.hkl

Supporting information file. DOI: 10.1107/S2056989018018376/jj2206Isup3.cml

CCDC reference: 1887613

Additional supporting information:  crystallographic information; 3D view; checkCIF report

## Figures and Tables

**Table 1:** Hydrogen-bond geometry (Å, °) *Cg*1 is the centroid of the C1–C6 ring.

*D*—H⋯*A*	*D*—H	H⋯*A*	*D*⋯*A*	*D*—H⋯*A*
N5—H5′⋯O1	0.88 (1)	1.99 (1)	2.8639 (19)	174 (2)
N2—H2′⋯O2	0.88 (1)	1.93 (1)	2.808 (2)	176 (2)
N3—H3′⋯N1	0.87 (1)	2.13 (2)	2.6146 (18)	115 (1)
N6—H6′⋯N4	0.87 (1)	2.17 (2)	2.6261 (19)	112 (2)
C13—H13⋯O2^i^	0.93	2.64	3.476 (2)	149
C32—H32⋯*Cg*1^ii^	0.93	2.79	3.518 (2)	136

Symmetry codes: (i) 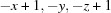; (ii) 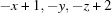.

**Table 2:** Experimental details

Crystal data	
Chemical formula	C_17_H_17_N_3_O
*M*_r_	279.33
Crystal system, space group	Triclinic, *P* 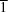
Temperature (K)	296
*a*, *b*, *c* (Å)	10.2140 (6), 10.5133 (8), 15.3297 (10)
α, β, γ (°)	106.652 (3), 99.111 (3), 97.416 (4)
*V* (Å^3^)	1530.51 (18)
*Z*	4
Radiation type	Mo *K*α
μ (mm^−1^)	0.08
Crystal size (mm)	0.60 × 0.50 × 0.50

Data collection	
Diffractometer	Bruker Kappa APEXII CCD
Absorption correction	Multi-scan (*SADABS*; Bruker, 2004 ▸)
*T*_min_, *T*_max_	0.939, 0.948
No. of measured, independent and observed [*I* > 2σ(*I*)] reflections	12172, 7346, 4038
*R*_int_	0.019
(sin θ/λ)_max_ (Å^−1^)	0.669

Refinement	
*R*[*F* ^2^ > 2σ(*F* ^2^)], *wR*(*F* ^2^), *S*	0.051, 0.175, 0.95
No. of reflections	7346
No. of parameters	397
No. of restraints	4
H-atom treatment	H atoms treated by a mixture of independent and constrained refinement
Δρ_max_, Δρ_min_ (e Å^−3^)	0.16, −0.23

Computer programs: *APEX2*, *SAINT* and *XPREP* (Bruker, 2004 ▸), *SHELXL2014* (Sheldrick, 2015 ▸), *SHELXS97* (Sheldrick, 2008 ▸), *ORTEP-3 for Windows* (Farrugia, 2012 ▸), *DIAMOND* (Brandenburg, 2010 ▸) and *publCIF* (Westrip, 2010 ▸).

## supplementary crystallographic information

### Crystal data

**Table:**

C_17_H_17_N_3_O	*Z* = 4
*M**_r_* = 279.33	*F*(000) = 592
Triclinic, *P*1	*D*_x_ = 1.212 Mg m^−^^3^
*a* = 10.2140 (6) Å	Mo *K*α radiation, λ = 0.71073 Å
*b* = 10.5133 (8) Å	Cell parameters from 3309 reflections
*c* = 15.3297 (10) Å	θ = 2.6–27.7°
α = 106.652 (3)°	µ = 0.08 mm^−^^1^
β = 99.111 (3)°	*T* = 296 K
γ = 97.416 (4)°	Block, colorless
*V* = 1530.51 (18) Å^3^	0.60 × 0.50 × 0.50 mm

### Data collection

**Table:**

Bruker Kappa APEXII CCD diffractometer	7346 independent reflections
Radiation source: fine-focus sealed tube	4038 reflections with *I* > 2σ(*I*)
Graphite monochromator	*R*_int_ = 0.019
ω and φ scan	θ_max_ = 28.4°, θ_min_ = 2.2°
Absorption correction: multi-scan (SADABS; Bruker, 2004)	*h* = −13→12
*T*_min_ = 0.939, *T*_max_ = 0.948	*k* = −13→13
12172 measured reflections	*l* = −20→15

### Refinement

**Table:**

Refinement on *F*^2^	4 restraints
Least-squares matrix: full	Hydrogen site location: mixed
*R*[*F*^2^ > 2σ(*F*^2^)] = 0.051	H atoms treated by a mixture of independent and constrained refinement
*wR*(*F*^2^) = 0.175	*w* = 1/[σ^2^(*F*_o_^2^) + (0.1017*P*)^2^] where *P* = (*F*_o_^2^ + 2*F*_c_^2^)/3
*S* = 0.95	(Δ/σ)_max_ < 0.001
7346 reflections	Δρ_max_ = 0.16 e Å^−^^3^
397 parameters	Δρ_min_ = −0.23 e Å^−^^3^

### Special details

**Table:**

Geometry. All esds (except the esd in the dihedral angle between two l.s. planes) are estimated using the full covariance matrix. The cell esds are taken into account individually in the estimation of esds in distances, angles and torsion angles; correlations between esds in cell parameters are only used when they are defined by crystal symmetry. An approximate (isotropic) treatment of cell esds is used for estimating esds involving l.s. planes.

### Fractional atomic coordinates and isotropic or equivalent isotropic displacement parameters (Å^2^)

**Table:**

	*x*	*y*	*z*	*U*_iso_*/*U*_eq_	
C1	0.88173 (19)	−0.1561 (2)	0.82683 (13)	0.0630 (5)	
H1	0.8824	−0.0637	0.8438	0.076*	
C2	0.9866 (2)	−0.2034 (3)	0.86846 (15)	0.0723 (6)	
H2	1.0576	−0.1430	0.9125	0.087*	
C3	0.9863 (2)	−0.3387 (3)	0.84506 (16)	0.0753 (6)	
H3	1.0568	−0.3706	0.8732	0.090*	
C4	0.8822 (2)	−0.4271 (2)	0.78030 (15)	0.0734 (6)	
H4	0.8818	−0.5194	0.7647	0.088*	
C5	0.7773 (2)	−0.3802 (2)	0.73766 (13)	0.0613 (5)	
H5	0.7073	−0.4415	0.6933	0.074*	
C6	0.77527 (16)	−0.2438 (2)	0.76005 (12)	0.0497 (4)	
C7	0.66419 (17)	−0.18955 (19)	0.71894 (12)	0.0502 (4)	
H7	0.6304	−0.1251	0.7604	0.060*	
C8	0.60596 (15)	−0.22156 (18)	0.62933 (11)	0.0442 (4)	
C9	0.64815 (19)	−0.3180 (2)	0.55182 (13)	0.0611 (5)	
H9A	0.7350	−0.3360	0.5739	0.092*	
H9B	0.6534	−0.2795	0.5025	0.092*	
H9C	0.5834	−0.4008	0.5290	0.092*	
C10	0.49813 (15)	−0.15284 (18)	0.60452 (11)	0.0448 (4)	
H10	0.4637	−0.0979	0.6512	0.054*	
C11	0.29530 (15)	−0.10122 (17)	0.41634 (11)	0.0438 (4)	
C12	0.32548 (15)	−0.18810 (17)	0.25412 (11)	0.0447 (4)	
C13	0.42849 (18)	−0.2055 (2)	0.20670 (13)	0.0649 (6)	
H13	0.5143	−0.2049	0.2382	0.078*	
C14	0.4051 (2)	−0.2239 (3)	0.11315 (14)	0.0850 (8)	
H14	0.4754	−0.2355	0.0815	0.102*	
C15	0.2788 (2)	−0.2254 (3)	0.06550 (14)	0.0777 (7)	
H15	0.2634	−0.2373	0.0020	0.093*	
C16	0.17664 (19)	−0.2093 (2)	0.11236 (13)	0.0640 (5)	
H16	0.0907	−0.2110	0.0804	0.077*	
C17	0.19852 (16)	−0.1905 (2)	0.20641 (12)	0.0541 (5)	
H17	0.1278	−0.1794	0.2377	0.065*	
C18	−0.33204 (19)	0.2586 (2)	0.20723 (13)	0.0665 (6)	
H18	−0.3167	0.1721	0.1801	0.080*	
C19	−0.4276 (2)	0.3093 (3)	0.15904 (15)	0.0787 (7)	
H19	−0.4753	0.2573	0.0999	0.094*	
C20	−0.4518 (2)	0.4367 (3)	0.19863 (16)	0.0793 (7)	
H20	−0.5171	0.4706	0.1670	0.095*	
C21	−0.3796 (2)	0.5128 (3)	0.28446 (17)	0.0769 (6)	
H21	−0.3953	0.5993	0.3110	0.092*	
C22	−0.2836 (2)	0.4633 (2)	0.33250 (14)	0.0643 (5)	
H22	−0.2347	0.5174	0.3908	0.077*	
C23	−0.25859 (16)	0.3344 (2)	0.29541 (12)	0.0507 (4)	
C24	−0.15906 (16)	0.27357 (19)	0.34168 (12)	0.0498 (4)	
H24	−0.1223	0.2101	0.3017	0.060*	
C25	−0.11244 (15)	0.29476 (17)	0.43239 (11)	0.0431 (4)	
C26	−0.15538 (17)	0.38927 (19)	0.51113 (12)	0.0516 (4)	
H26A	−0.0976	0.4761	0.5297	0.077*	
H26B	−0.1493	0.3543	0.5627	0.077*	
H26C	−0.2469	0.3982	0.4916	0.077*	
C27	−0.01521 (15)	0.21447 (17)	0.45568 (11)	0.0451 (4)	
H27	0.0122	0.1537	0.4079	0.054*	
C28	0.17376 (15)	0.13993 (19)	0.63940 (12)	0.0474 (4)	
C29	0.17182 (15)	0.24127 (19)	0.80442 (12)	0.0482 (4)	
C30	0.1829 (2)	0.3675 (2)	0.86669 (14)	0.0740 (6)	
H30	0.1683	0.4397	0.8452	0.089*	
C31	0.2159 (2)	0.3874 (3)	0.96110 (15)	0.0878 (7)	
H31	0.2219	0.4729	1.0029	0.105*	
C32	0.23933 (19)	0.2836 (3)	0.99329 (15)	0.0733 (6)	
H32	0.2616	0.2974	1.0569	0.088*	
C33	0.2302 (2)	0.1594 (3)	0.93208 (15)	0.0766 (7)	
H33	0.2476	0.0882	0.9540	0.092*	
C34	0.1954 (2)	0.1371 (2)	0.83737 (14)	0.0675 (5)	
H34	0.1880	0.0510	0.7961	0.081*	
N1	0.44999 (12)	−0.16681 (14)	0.51975 (9)	0.0447 (3)	
N2	0.34882 (14)	−0.09748 (16)	0.50394 (10)	0.0485 (4)	
N3	0.35427 (14)	−0.17255 (17)	0.34979 (10)	0.0536 (4)	
N4	0.03382 (12)	0.22481 (14)	0.53988 (9)	0.0455 (3)	
N5	0.12237 (14)	0.14139 (16)	0.55249 (10)	0.0516 (4)	
N6	0.13105 (14)	0.22397 (17)	0.70885 (10)	0.0538 (4)	
O1	0.20160 (11)	−0.04300 (13)	0.40141 (8)	0.0525 (3)	
O2	0.25451 (13)	0.06620 (15)	0.65140 (8)	0.0648 (4)	
H3'	0.4260 (12)	−0.1964 (17)	0.3742 (11)	0.049 (5)*	
H5'	0.1507 (17)	0.0898 (17)	0.5059 (9)	0.060 (6)*	
H6'	0.080 (2)	0.276 (2)	0.6905 (15)	0.087 (7)*	
H2'	0.3176 (16)	−0.0501 (17)	0.5509 (9)	0.055 (5)*	

### Atomic displacement parameters (Å^2^)

**Table:**

	*U*^11^	*U*^22^	*U*^33^	*U*^12^	*U*^13^	*U*^23^
C1	0.0670 (11)	0.0636 (14)	0.0591 (11)	0.0160 (10)	0.0032 (9)	0.0238 (10)
C2	0.0595 (11)	0.0850 (18)	0.0701 (13)	0.0139 (11)	−0.0047 (10)	0.0299 (13)
C3	0.0660 (12)	0.0986 (19)	0.0719 (14)	0.0403 (12)	0.0071 (11)	0.0358 (14)
C4	0.0886 (14)	0.0696 (15)	0.0705 (14)	0.0405 (12)	0.0118 (12)	0.0262 (12)
C5	0.0664 (11)	0.0615 (14)	0.0568 (11)	0.0221 (9)	0.0036 (9)	0.0204 (10)
C6	0.0519 (9)	0.0611 (12)	0.0446 (10)	0.0185 (8)	0.0118 (8)	0.0253 (9)
C7	0.0561 (10)	0.0558 (12)	0.0463 (10)	0.0227 (8)	0.0123 (8)	0.0209 (9)
C8	0.0415 (8)	0.0493 (10)	0.0467 (10)	0.0124 (7)	0.0108 (7)	0.0198 (8)
C9	0.0615 (11)	0.0734 (14)	0.0521 (11)	0.0290 (10)	0.0109 (8)	0.0186 (10)
C10	0.0455 (8)	0.0494 (11)	0.0446 (9)	0.0136 (7)	0.0116 (7)	0.0192 (8)
C11	0.0416 (8)	0.0477 (10)	0.0457 (9)	0.0108 (7)	0.0081 (7)	0.0197 (8)
C12	0.0453 (9)	0.0457 (10)	0.0419 (9)	0.0117 (7)	0.0044 (7)	0.0128 (8)
C13	0.0478 (10)	0.0906 (16)	0.0518 (11)	0.0219 (10)	0.0054 (8)	0.0141 (11)
C14	0.0620 (12)	0.138 (2)	0.0514 (12)	0.0239 (13)	0.0164 (10)	0.0196 (14)
C15	0.0725 (13)	0.113 (2)	0.0420 (11)	0.0223 (12)	0.0025 (10)	0.0188 (12)
C16	0.0545 (10)	0.0749 (15)	0.0532 (11)	0.0158 (9)	−0.0066 (9)	0.0134 (10)
C17	0.0432 (9)	0.0663 (13)	0.0519 (11)	0.0134 (8)	0.0044 (8)	0.0185 (9)
C18	0.0621 (11)	0.0881 (17)	0.0470 (11)	0.0220 (10)	0.0057 (9)	0.0169 (11)
C19	0.0634 (12)	0.120 (2)	0.0508 (12)	0.0199 (13)	−0.0005 (9)	0.0295 (13)
C20	0.0599 (12)	0.116 (2)	0.0783 (16)	0.0254 (13)	0.0060 (11)	0.0555 (16)
C21	0.0739 (13)	0.0754 (16)	0.0897 (16)	0.0222 (11)	0.0032 (12)	0.0422 (14)
C22	0.0677 (12)	0.0597 (14)	0.0642 (12)	0.0129 (9)	−0.0033 (9)	0.0257 (11)
C23	0.0463 (9)	0.0648 (13)	0.0449 (10)	0.0096 (8)	0.0081 (7)	0.0240 (9)
C24	0.0504 (9)	0.0522 (11)	0.0458 (10)	0.0135 (8)	0.0078 (7)	0.0133 (8)
C25	0.0403 (8)	0.0432 (10)	0.0439 (9)	0.0064 (7)	0.0046 (7)	0.0137 (8)
C26	0.0530 (9)	0.0535 (12)	0.0508 (10)	0.0179 (8)	0.0109 (8)	0.0163 (9)
C27	0.0445 (8)	0.0455 (10)	0.0431 (9)	0.0114 (7)	0.0069 (7)	0.0103 (8)
C28	0.0397 (8)	0.0527 (11)	0.0461 (10)	0.0114 (7)	0.0022 (7)	0.0120 (8)
C29	0.0404 (8)	0.0587 (12)	0.0454 (9)	0.0135 (7)	0.0081 (7)	0.0147 (9)
C30	0.0992 (15)	0.0679 (15)	0.0585 (13)	0.0323 (12)	0.0211 (11)	0.0154 (11)
C31	0.1139 (19)	0.0819 (18)	0.0553 (14)	0.0197 (14)	0.0196 (13)	0.0005 (13)
C32	0.0642 (12)	0.104 (2)	0.0472 (12)	0.0100 (12)	0.0097 (9)	0.0199 (13)
C33	0.0860 (15)	0.0902 (19)	0.0625 (14)	0.0214 (13)	0.0101 (11)	0.0380 (14)
C34	0.0845 (13)	0.0593 (14)	0.0558 (12)	0.0146 (10)	0.0066 (10)	0.0174 (10)
N1	0.0417 (7)	0.0494 (9)	0.0485 (8)	0.0147 (6)	0.0088 (6)	0.0214 (7)
N2	0.0490 (8)	0.0593 (10)	0.0444 (8)	0.0231 (7)	0.0120 (6)	0.0207 (7)
N3	0.0499 (8)	0.0735 (11)	0.0438 (8)	0.0295 (7)	0.0071 (6)	0.0216 (8)
N4	0.0417 (7)	0.0472 (9)	0.0465 (8)	0.0127 (6)	0.0045 (6)	0.0136 (7)
N5	0.0512 (8)	0.0588 (10)	0.0441 (9)	0.0247 (7)	0.0042 (7)	0.0119 (8)
N6	0.0547 (8)	0.0642 (11)	0.0449 (8)	0.0263 (7)	0.0072 (7)	0.0159 (8)
O1	0.0492 (6)	0.0641 (9)	0.0501 (7)	0.0244 (6)	0.0089 (5)	0.0218 (6)
O2	0.0679 (8)	0.0774 (10)	0.0485 (7)	0.0406 (7)	0.0013 (6)	0.0124 (7)

### Geometric parameters (Å, º)

**Table:**

C1—C2	1.381 (2)	C19—C20	1.375 (3)
C1—C6	1.389 (3)	C19—H19	0.9300
C1—H1	0.9300	C20—C21	1.361 (3)
C2—C3	1.362 (3)	C20—H20	0.9300
C2—H2	0.9300	C21—C22	1.378 (2)
C3—C4	1.364 (3)	C21—H21	0.9300
C3—H3	0.9300	C22—C23	1.386 (3)
C4—C5	1.385 (2)	C22—H22	0.9300
C4—H4	0.9300	C23—C24	1.464 (2)
C5—C6	1.379 (3)	C24—C25	1.340 (2)
C5—H5	0.9300	C24—H24	0.9300
C6—C7	1.469 (2)	C25—C27	1.450 (2)
C7—C8	1.334 (2)	C25—C26	1.493 (2)
C7—H7	0.9300	C26—H26A	0.9600
C8—C10	1.453 (2)	C26—H26B	0.9600
C8—C9	1.487 (2)	C26—H26C	0.9600
C9—H9A	0.9600	C27—N4	1.2766 (19)
C9—H9B	0.9600	C27—H27	0.9300
C9—H9C	0.9600	C28—O2	1.2302 (18)
C10—N1	1.273 (2)	C28—N6	1.346 (2)
C10—H10	0.9300	C28—N5	1.358 (2)
C11—O1	1.2269 (17)	C29—C34	1.363 (3)
C11—N2	1.355 (2)	C29—C30	1.373 (3)
C11—N3	1.356 (2)	C29—N6	1.410 (2)
C12—C13	1.373 (2)	C30—C31	1.380 (3)
C12—C17	1.378 (2)	C30—H30	0.9300
C12—N3	1.406 (2)	C31—C32	1.354 (3)
C13—C14	1.368 (3)	C31—H31	0.9300
C13—H13	0.9300	C32—C33	1.353 (3)
C14—C15	1.374 (3)	C32—H32	0.9300
C14—H14	0.9300	C33—C34	1.382 (3)
C15—C16	1.359 (3)	C33—H33	0.9300
C15—H15	0.9300	C34—H34	0.9300
C16—C17	1.375 (3)	N1—N2	1.3691 (17)
C16—H16	0.9300	N2—H2'	0.881 (9)
C17—H17	0.9300	N3—H3'	0.868 (9)
C18—C19	1.383 (3)	N4—N5	1.3679 (18)
C18—C23	1.390 (3)	N5—H5'	0.879 (9)
C18—H18	0.9300	N6—H6'	0.872 (9)
			
C2—C1—C6	121.3 (2)	C21—C20—H20	120.2
C2—C1—H1	119.4	C19—C20—H20	120.2
C6—C1—H1	119.4	C20—C21—C22	120.8 (2)
C3—C2—C1	120.0 (2)	C20—C21—H21	119.6
C3—C2—H2	120.0	C22—C21—H21	119.6
C1—C2—H2	120.0	C21—C22—C23	121.1 (2)
C2—C3—C4	119.84 (18)	C21—C22—H22	119.5
C2—C3—H3	120.1	C23—C22—H22	119.5
C4—C3—H3	120.1	C22—C23—C18	117.31 (16)
C3—C4—C5	120.4 (2)	C22—C23—C24	124.73 (16)
C3—C4—H4	119.8	C18—C23—C24	117.95 (18)
C5—C4—H4	119.8	C25—C24—C23	130.34 (16)
C6—C5—C4	120.90 (19)	C25—C24—H24	114.8
C6—C5—H5	119.6	C23—C24—H24	114.8
C4—C5—H5	119.6	C24—C25—C27	116.69 (15)
C5—C6—C1	117.52 (16)	C24—C25—C26	125.97 (15)
C5—C6—C7	122.88 (17)	C27—C25—C26	117.30 (14)
C1—C6—C7	119.57 (17)	C25—C26—H26A	109.5
C8—C7—C6	127.81 (16)	C25—C26—H26B	109.5
C8—C7—H7	116.1	H26A—C26—H26B	109.5
C6—C7—H7	116.1	C25—C26—H26C	109.5
C7—C8—C10	118.17 (15)	H26A—C26—H26C	109.5
C7—C8—C9	124.61 (15)	H26B—C26—H26C	109.5
C10—C8—C9	117.15 (14)	N4—C27—C25	121.85 (15)
C8—C9—H9A	109.5	N4—C27—H27	119.1
C8—C9—H9B	109.5	C25—C27—H27	119.1
H9A—C9—H9B	109.5	O2—C28—N6	123.87 (15)
C8—C9—H9C	109.5	O2—C28—N5	120.66 (16)
H9A—C9—H9C	109.5	N6—C28—N5	115.48 (14)
H9B—C9—H9C	109.5	C34—C29—C30	118.95 (17)
N1—C10—C8	120.96 (15)	C34—C29—N6	122.71 (17)
N1—C10—H10	119.5	C30—C29—N6	118.29 (17)
C8—C10—H10	119.5	C29—C30—C31	120.2 (2)
O1—C11—N2	120.94 (15)	C29—C30—H30	119.9
O1—C11—N3	124.61 (14)	C31—C30—H30	119.9
N2—C11—N3	114.45 (13)	C32—C31—C30	120.5 (2)
C13—C12—C17	119.33 (16)	C32—C31—H31	119.8
C13—C12—N3	117.83 (13)	C30—C31—H31	119.8
C17—C12—N3	122.81 (15)	C33—C32—C31	119.5 (2)
C14—C13—C12	120.14 (16)	C33—C32—H32	120.3
C14—C13—H13	119.9	C31—C32—H32	120.3
C12—C13—H13	119.9	C32—C33—C34	120.8 (2)
C13—C14—C15	120.64 (19)	C32—C33—H33	119.6
C13—C14—H14	119.7	C34—C33—H33	119.6
C15—C14—H14	119.7	C29—C34—C33	120.1 (2)
C16—C15—C14	119.16 (18)	C29—C34—H34	120.0
C16—C15—H15	120.4	C33—C34—H34	120.0
C14—C15—H15	120.4	C10—N1—N2	116.30 (14)
C15—C16—C17	120.91 (16)	C11—N2—N1	120.63 (14)
C15—C16—H16	119.5	C11—N2—H2'	119.3 (11)
C17—C16—H16	119.5	N1—N2—H2'	120.1 (11)
C16—C17—C12	119.81 (17)	C11—N3—C12	127.30 (13)
C16—C17—H17	120.1	C11—N3—H3'	111.3 (11)
C12—C17—H17	120.1	C12—N3—H3'	120.4 (11)
C19—C18—C23	121.3 (2)	C27—N4—N5	116.14 (14)
C19—C18—H18	119.4	C28—N5—N4	120.26 (15)
C23—C18—H18	119.4	C28—N5—H5'	117.5 (12)
C20—C19—C18	119.9 (2)	N4—N5—H5'	122.2 (12)
C20—C19—H19	120.0	C28—N6—C29	125.66 (14)
C18—C19—H19	120.0	C28—N6—H6'	113.7 (15)
C21—C20—C19	119.53 (19)	C29—N6—H6'	120.3 (15)
			
C6—C1—C2—C3	−0.8 (3)	C22—C23—C24—C25	29.1 (3)
C1—C2—C3—C4	0.1 (3)	C18—C23—C24—C25	−152.1 (2)
C2—C3—C4—C5	0.5 (4)	C23—C24—C25—C27	178.40 (17)
C3—C4—C5—C6	−0.4 (3)	C23—C24—C25—C26	0.9 (3)
C4—C5—C6—C1	−0.2 (3)	C24—C25—C27—N4	−178.73 (16)
C4—C5—C6—C7	−177.99 (19)	C26—C25—C27—N4	−1.0 (2)
C2—C1—C6—C5	0.8 (3)	C34—C29—C30—C31	−0.8 (3)
C2—C1—C6—C7	178.68 (18)	N6—C29—C30—C31	176.69 (19)
C5—C6—C7—C8	−47.8 (3)	C29—C30—C31—C32	0.9 (4)
C1—C6—C7—C8	134.4 (2)	C30—C31—C32—C33	−0.1 (4)
C6—C7—C8—C10	−179.13 (17)	C31—C32—C33—C34	−1.0 (3)
C6—C7—C8—C9	−2.4 (3)	C30—C29—C34—C33	−0.2 (3)
C7—C8—C10—N1	171.39 (16)	N6—C29—C34—C33	−177.57 (17)
C9—C8—C10—N1	−5.6 (3)	C32—C33—C34—C29	1.1 (3)
C17—C12—C13—C14	0.6 (3)	C8—C10—N1—N2	−179.88 (14)
N3—C12—C13—C14	178.7 (2)	O1—C11—N2—N1	177.56 (15)
C12—C13—C14—C15	−0.1 (4)	N3—C11—N2—N1	−2.8 (2)
C13—C14—C15—C16	−0.4 (4)	C10—N1—N2—C11	177.09 (15)
C14—C15—C16—C17	0.6 (4)	O1—C11—N3—C12	3.3 (3)
C15—C16—C17—C12	−0.1 (3)	N2—C11—N3—C12	−176.34 (17)
C13—C12—C17—C16	−0.4 (3)	C13—C12—N3—C11	149.8 (2)
N3—C12—C17—C16	−178.43 (18)	C17—C12—N3—C11	−32.2 (3)
C23—C18—C19—C20	−0.4 (3)	C25—C27—N4—N5	178.64 (15)
C18—C19—C20—C21	1.2 (4)	O2—C28—N5—N4	−179.26 (16)
C19—C20—C21—C22	−0.7 (4)	N6—C28—N5—N4	0.2 (2)
C20—C21—C22—C23	−0.7 (3)	C27—N4—N5—C28	−176.31 (16)
C21—C22—C23—C18	1.4 (3)	O2—C28—N6—C29	0.5 (3)
C21—C22—C23—C24	−179.73 (19)	N5—C28—N6—C29	−179.02 (16)
C19—C18—C23—C22	−0.9 (3)	C34—C29—N6—C28	−37.1 (3)
C19—C18—C23—C24	−179.82 (19)	C30—C29—N6—C28	145.5 (2)

### Hydrogen-bond geometry (Å, º)

*Cg*1 is the centroid of the C1–C6 ring.

**Table:**

*D*—H···*A*	*D*—H	H···*A*	*D*···*A*	*D*—H···*A*
N5—H5′···O1	0.88 (1)	1.99 (1)	2.8639 (19)	174 (2)
N2—H2′···O2	0.88 (1)	1.93 (1)	2.808 (2)	176 (2)
N3—H3′···N1	0.87 (1)	2.13 (2)	2.6146 (18)	115 (1)
N6—H6′···N4	0.87 (1)	2.17 (2)	2.6261 (19)	112 (2)
C13—H13···O2^i^	0.93	2.64	3.476 (2)	149
C32—H32···*Cg*1^ii^	0.93	2.79	3.518 (2)	136

Symmetry codes: (i) −*x*+1, −*y*, −*z*+1; (ii) −*x*+1, −*y*, −*z*+2.
